# Morphology-Guided Screening of PLA/Cellulose Biocomposites for Melt-Spun Monofilaments: Micronized Eucalyptus Kraft Pulp Versus Microcrystalline Cellulose

**DOI:** 10.3390/polym18141787

**Published:** 2026-07-22

**Authors:** Mário Pinto, Susana Gomes, Manuel Vieira, Juliana Silva, Alexandre Gaspar, Bruno F. A. Valente, Tomás Duarte, Paulo F. Teixeira

**Affiliations:** 1 CeNTI—Centre for Nanotechnology and Advanced Materials, R. Fernando Mesquita 2785, 4760-034 Vila Nova de Famalicão, Portugal; mpinto@centi.pt (M.P.); sgomes@centi.pt (S.G.); mvieira@centi.pt (M.V.); jssilva@centi.pt (J.S.); 2 RAIZ—Forest and Paper Research Institute, Quinta de S. Francisco, Rua José Estevão (EN 230-1), 3800-783 Aveiro, Portugal; alexandre.gaspar@thenavigatorcompany.com (A.G.); bruno.valente@thenavigatorcompany.com (B.F.A.V.); tomas.a.duarte@thenavigatorcompany.com (T.D.)

**Keywords:** poly(lactic acid), bleached eucalyptus kraft pulp, microcrystalline cellulose, cellulose morphology, biocomposites, melt compounding, melt spinning

## Abstract

Poly(lactic acid) (PLA) is a promising bio-based polymer for melt-spun fibres, but its low melt strength, brittleness and narrow processing window limit broader use. This study evaluates PLA/cellulose biocomposites for monofilament melt spinning by comparing micronized bleached Eucalyptus kraft pulp-derived fibres (P200) with microcrystalline cellulose (MCC) as a particulate reference, within a fixed compatibilizer/processing-aid formulation. Composites containing different cellulose contents were prepared by melt compounding and assessed by thermal analysis, oscillatory rheology, mechanical testing and fracture morphology; selected 5 wt.% formulations were then processed into monofilaments. Cellulose incorporation increased the onset degradation temperature of the PLA reference, while the rheological and mechanical responses depended strongly on reinforcement morphology. P200 promoted stronger low-frequency melt structuring, stiffness and crystallization tendency, but also a greater reduction in ductility, with notched impact strength decreasing significantly only at the highest P200 loading. MCC produced a more moderate composite-level response. Stable continuous monofilaments were obtained from both selected cellulose-containing formulations, although neither matched the drawable window or draw-induced mechanical development of the PLA reference formulation. Among the cellulose-containing systems, the 5 wt.% MCC formulation retained the more favourable balance between reinforcement and melt-spinning processability.

## 1. Introduction

The increasing concern about environmental pollution, plastic waste, and depletion of fossil resources is driving the development of biodegradable and bio-based polymers for technical textiles, packaging, and health-related applications [1,2,3,4]. These application areas also represent large industrial sectors, reinforcing the need for scalable bio-based materials compatible with established polymer-processing routes [5,6,7]. Among the biodegradable and bio-based polymers being explored for such applications, poly(lactic acid) (PLA) is one of the most prominent candidates due to its derivation from renewable feedstocks, its industrial compostability under controlled composting conditions, and its compatibility with conventional polymer-processing technologies [8,9]. Several recent reviews have comprehensively summarized the development of biodegradable polymer fibres, including PLA-based systems, and their processing by melt spinning [10,11], highlighting both opportunities and persistent challenges in achieving industrial-scale performance. Melt spinning, a solvent-free, cost-effective, and scalable technique, has become a widely used method for producing continuous polymer filaments [12,13]. This method is particularly attractive for PLA-based fibres, as it aligns with the sustainability and circular-economy principles associated with bio-based polymers.

However, despite its promise, the broader use of PLA in fibre applications remains limited by its relatively low melt strength, brittleness, and narrow processing window, which may restrict its spinnability and reduce the mechanical performance of the resulting filaments [11,14,15]. To address these shortcomings, researchers have explored strategies such as the incorporation of chain extenders, flexible polyesters like poly(butylene succinate) (PBS) or poly(butylene adipate-co-terephthalate) (PBAT), and low-molecular-weight plasticizers [16,17,18,19,20]. These modifications can improve ductility and processability, but they often compromise stiffness, thermal stability, or biodegradation rate, motivating the search for alternative reinforcement strategies that retain high mechanical strength while maintaining the biodegradability of the PLA-based matrix [11].

Reinforcing PLA with natural fibres has emerged as an effective and sustainable approach to simultaneously improve stiffness and strength, reduce material cost, and increase the overall bio-based content of the product [21,22]. Recent reviews have highlighted the rapid progress of natural fibre-reinforced thermoplastics, emphasizing their mechanical performance, interfacial adhesion strategies, and sustainability benefits [23,24,25]. These works underscore the widespread investigation of cellulose-based reinforcements, such as wood pulp, flax, hemp, and jute, due to their abundance, renewability, and favourable stiffness-to-weight ratio. Within this broader context, bleached Eucalyptus kraft pulp (BEKP) has been explored as a commercially available cellulosic reinforcement for PLA-based composites, making it a relevant candidate for further study in melt-spun PLA systems [26].

Several studies have demonstrated the reinforcing potential of cellulose fibres in thermoplastic matrices. Classical work by Bledzki and Gassan [27] established that cellulose fibres can substantially increase the tensile modulus and yield strength of polymers, while maintaining low density. More recently, Valente et al. [28] used micronized BEKP fibres to reinforce PLA and PHB matrices, reporting simultaneous increases in tensile and flexural moduli, together with improved composite homogeneity and melt-flow behaviour relative to non-micronized BEKP. Zarna et al. [29] reinforced PLA with thermomechanical wood pulp fibres and found that lowering the processing temperature from 200 °C to 180 °C improved final properties, consistent with reduced thermal damage during compounding. Nanthananon et al. [30] incorporated eucalypt pulp into PLA and PBS using reactive compatibilizers (Joncryl, peroxide), reporting improved modulus and enhanced fibre–matrix compatibility at low reactive-agent contents. These findings confirm the potential of BEKP or similar fibres in thermoplastics and illustrate the practical challenges that accompany their melt processing.

Despite these promising findings, integrating cellulose fibres, particularly micronized BEKP-derived fibres, into hydrophobic thermoplastics such as PLA remains challenging. The hydrophilic character and moisture uptake of cellulose promote poor interfacial adhesion with the PLA matrix, leading to fibre pull-out, voids, and inefficient stress transfer [27,31]. Furthermore, cellulose’s thermal sensitivity and moisture absorption require careful processing control to avoid hydrolysis of PLA and fibre degradation during extrusion or spinning [12,29,32]. These issues are magnified in melt spinning, where consistent melt flow and fibre homogeneity are crucial to produce defect-free, continuous filaments. Achieving a homogeneous, well-bonded, and adequately dried PLA/cellulose blend with sufficient melt strength is therefore a prerequisite for successful fibre spinning.

Although interest in cellulose-reinforced PLA systems is growing, few studies have systematically assessed the melt-spinning viability of industrially available micronized pulp-derived fibres. Previous work on melt-spun PLA/cellulose multifilaments has shown that cellulose morphology can strongly affect fibre formation, with MCC-containing formulations displaying restricted drawability and poorer dispersion than nanocellulose-containing systems [33]. Nanoscale cellulose reinforcements such as nanocrystals (CNCs) and nanofibrils (CNFs) are widely studied for PLA nanocomposites [34,35,36]. The effective reinforcement of such systems depends not only on intrinsic stiffness and aspect ratio, but also on interfacial interactions and network formation, as illustrated by studies on CNC interactions with lignocellulosic polymers and CNC/lignin-containing cellulose-based composites [37,38]. However, their industrial application remains limited by high cost, restricted availability, and still-limited commercial uptake [39,40]. In addition, their melt processing is challenged by poor dispersion, increased melt viscosity, and sensitivity to thermal degradation [32], despite their excellent intrinsic mechanical properties and high reinforcing potential [39]. These observations highlight the importance of reinforcement morphology in PLA fibre processing, but do not address the behaviour of micronized pulp-derived microfibres as melt-spinning precursors.

Thus, nanoscale fillers are not yet widely adopted in large-scale melt spinning. To place the performance of micronized BEKP-derived fibres within an industrially relevant context and to compare a fibrous micro-scale reinforcement with a particulate reference, microcrystalline cellulose (MCC) was selected as a commercially available micro-scale reference filler. MCC has also been investigated in melt-processed PLA-based composites, where its incorporation was shown to affect rheological, thermal, and mechanical behaviour, making it a useful benchmark for comparison with micronized BEKP-derived fibres [41]. Its contrasting morphology allows direct evaluation of how reinforcement geometry influences dispersion, rheology, and filament integrity under identical processing conditions. Previous studies have addressed adjacent aspects of this problem. Aouat et al. investigated melt-spun PLA/cellulose multifilaments containing MCC or cellulose nanowhiskers and reported restricted drawability and poorer dispersion for MCC-containing formulations [33]. Valente et al. evaluated micronized BEKP fibres in injection-moulded PLA composites and showed that pulp micronization improved composite homogeneity and melt-flow behaviour relative to non-micronized BEKP [28]. However, a direct comparison between industrially relevant fibrous and particulate cellulose morphologies within a common PLA formulation and morphology-guided screening framework remains limited, particularly regarding how composite-level differences translate into formulation selection and subsequent monofilament melt spinning. In this context, the present work compares micronized bleached eucalyptus kraft pulp-derived fibres (P200) and microcrystalline cellulose (MCC) in a fibre-grade PLA system containing a fixed compatibilizer/processing-aid package selected to support PLA/cellulose interfacial interaction and melt processability. This additive package was kept constant in all formulations and was not treated as an optimization variable, allowing the effect of cellulose morphology and content to be assessed within a common PLA-based processing platform. The materials were first evaluated at composite level in terms of thermal behaviour, rheology, mechanical performance, and fracture morphology, and the selected formulations were then processed into continuous monofilaments by melt spinning. The aim was therefore to assess how reinforcement morphology influences formulation screening and filament-processing response within this fixed PLA/additive formulation, rather than to establish a fully optimized spinning formulation.

## 2. Materials and Methods

### 2.1. Materials

Polylactic acid (PLA) Ingeo™ Biopolymer 6202D (NatureWorks LLC, Minnetonka, MN, USA) was used as the bio-based polymer matrix. This PLA grade is a thermoplastic fibre-grade resin, supplied in pellet form, with a melt flow index of 15–30 g/10 min (measured at 210 °C, 2.16 kg), specific gravity of ~1.24 and relative viscosity of 3.1. A specialized compatibilizer, SCONA TPPL 5112 PA (BYK-Chemie GmbH, Wesel, Germany), was added to improve interfacial adhesion between PLA and cellulose. According to the manufacturer, SCONA TPPL 5112 PA is a high-performance polymer modifier based on PLA functionalized with maleic anhydride, which acts as a coupling agent for filler and fibre reinforced PLA composites. Additionally, the acrylic processing aid Plastistrength^®^ 552 (Arkema, Colombes, France) was incorporated into the formulation. This high–molecular weight acrylic additive is recommended for PLA-based systems to enhance melt strength and improve processability, thereby broadening the processing window and promoting a more homogeneous dispersion of fillers. In the present study, SCONA TPPL 5112 PA and Plastistrength^®^ 552 were used at fixed contents in all PLA-based formulations to provide a common compatibilization and processing-aid platform. Therefore, the additive package was not varied as an experimental factor, allowing the influence of cellulose type and loading to be evaluated without changing the additive chemistry.

To develop biodegradable composites, the above PLA matrix and additives were combined with natural cellulose reinforcements. Bleached eucalyptus kraft pulp (BEKP) with a crystallinity index of 70.1% was kindly provided by the Navigator Company (Aveiro, Portugal). According to the product data sheet, the as-received BEKP consisted mainly of cellulose and hemicellulose (90 ± 4 wt.%) and water (10 ± 4 wt.%), with no impurities relevant for classification and labelling. The micronized fibres (P200) were obtained from the same BEKP using a drum milling equipment (Pallman Fine grinding PS, Zweibrücken, Germany) armed with knives. The BEKP was fed into the cutting chamber and cut repeatedly between the rotor knives and stator knives against each other until the material could pass through the screen insert. A 200 µm sieve mesh was used to produce fibres with an average length and width of 226 µm and 19.5 µm, respectively, as determined by image analysis [28]. As a micro-scale reference filler, commercial microcrystalline cellulose (MCC) Arbocel^®^ UFC100 (J. Rettenmaier & Söhne GmbH, Rosenberg, Germany) with an average particle size of approximately 10 µm was used as received.

### 2.2. Compounding and Processing of the Biocomposites

Prior to melt processing, all materials were dried at 80 °C for 4 h to minimize moisture-induced hydrolysis. This condition was selected to reduce residual moisture before melt compounding while limiting unnecessary thermal exposure of the cellulosic reinforcements. The compositions and sample codes (wt.% of total formulation) are summarized in Table 1. A PLA-based reference containing the same additive package, but no cellulose, was prepared and denoted as PLA-add.

Two compounding routes were used depending on the reinforcement. For the P200 series, a previously prepared PLA/P200 masterbatch (60/40 wt.%) was diluted with neat PLA and compounded together with 5 wt.% SCONA TPPL 5112 PA and 5 wt.% Plastistrength^®^ 552 to obtain final P200 contents of 2.5, 5, 10, and 20 wt.% (Table 1). For the MCC series, the composites were produced by direct melt compounding of microcrystalline cellulose (Arbocel^®^ UFC100) with neat PLA and the same additive package, yielding final MCC contents of 2.5, 5, and 10 wt.% (Table 1). The selected composition range provided a common 2.5–10 wt.% interval for the direct comparison between fibrous and particulate cellulose morphologies, while P200-20 was included as an additional high-loading formulation to examine the response of the micronized pulp-derived reinforcement. The different preparation routes reflected the handling characteristics of each cellulose type; however, all final formulations were subjected to the same twin-screw extrusion step prior to characterization and melt spinning.

Melt compounding of all final formulations, including PLA-add, was carried out at CeNTI (Vila Nova de Famalicão, Portugal) using a co-rotating twin-screw extruder (LabTwin 21, Rondol Industrie, Nancy, France; 21 mm; L/D 25). The barrel temperatures were set to 165 °C in the feeding zone and 170 °C in zones 2–4 and at the die, and the screw speed was fixed at 200 rpm. The extrudates were water-cooled and pelletized using an inline strand pelletizer (SP50 EN, Coperion, Offenbach, Germany). Test specimens for mechanical characterization were prepared by injection moulding (BOY XS, Dr. Boy GmbH & Co. KG, Neustadt-Fernthal, Germany) at 205 °C barrel temperature and 25 °C mould temperature. The injection pressure and injection speed were set to 750 bar and 50 mm s^−1^, respectively. After cavity filling, a packing pressure of 550 bar was applied for 7 s. The cooling time was 20 s.

### 2.3. Melt-Spun Biocomposites

For melt spinning, the selected formulations were pre-dried overnight at 60 °C and subsequently dried at 80 °C for 4 h to minimize moisture-induced hydrolysis. The residual moisture content of the dried pellets was measured immediately prior to processing by gravimetric loss-on-drying using a RADWAG MA 50/1.X2.A.WH (Radwag, Radom, Poland) moisture analyser, and values below 0.1 wt.% were obtained for all selected formulations. Melt spinning was carried out at CeNTI (Vila Nova de Famalicão, Portugal) using a Hills TRC system (Hills Inc., West Melbourne, FL, USA) equipped with a spinneret containing a single orifice of 1.5 mm diameter. The molten PLA-based formulations were fed to the spinneret at a nominal volumetric flow rate of 12 cm^3^ min^−1^. After extrusion, filament solidification was achieved by air cooling in a quenching zone with a height of 2.5 m.

**Figure 1:**
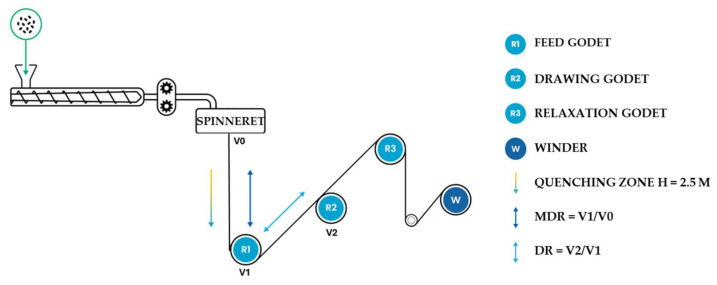
Schematic representation of the melt-spinning line used for monofilament production, showing the main stages of extrusion, air cooling, drawing, relaxation, and winding. V0 corresponds to the extrusion velocity at the spinneret exit, V1 to the feed godet velocity, V2 to the drawing godet velocity, and R3 to the relaxation godet. The yellow-to-blue gradient arrow represents the quenching and progressive cooling of the extruded filament, while the blue arrows indicate the drawing and relaxation directions.

The filaments were subsequently guided through a series of thermally controlled godets for drawing and relaxation. The first stage corresponded to melt drawing and was quantified by the melt draw ratio (MDR), defined as the ratio between the surface velocity of the feed godet (V1) and the extrusion velocity at the spinneret exit (V0). The subsequent stage corresponded to cold drawing and was quantified by the draw ratio (DR), defined as the ratio between the surface velocity of the drawing godet (V2) and that of the feed godet (V1). These parameters govern the extent of filament attenuation and molecular orientation and therefore strongly influence fibre properties such as tenacity, elongation at break, linear density, and diameter. After drawing, the filaments passed through a relaxation stage at the relaxation godet (R3) to relieve internal stresses and improve dimensional stability before winding. A schematic representation of the melt-spinning setup is shown in Figure 1. The processing conditions were adjusted according to the processability of each material and are discussed in the Results and Discussion section. The linear density of the melt-spun filaments was determined using a WRAP REEL ASPINO Elettronico—161M (Mesdan Lab, Puegnago del Garda, Italy), followed by mass measurement on an analytical balance. Filament diameter was measured using a Mitutoyo 547-526S thickness gauge (Mitutoyo, Kawasaki, Japan).

### 2.4. Characterization Methods

#### 2.4.1. Thermogravimetric Analysis

Thermogravimetric analysis (TGA) was performed using a TG 209 F1 Libra thermogravimetric analyser (NETZSCH-Gerätebau GmbH, Selb, Germany) equipped with a high-precision top-loading balance. Samples were placed in alumina crucibles and tested in accordance with ISO 11358-1:2022 [42]. Measurements were carried out under a nitrogen atmosphere with a gas flow rate of 50 mL min^−1^, using a heating rate of 10 °C min^−1^ over the temperature range from 30 to 700 °C. The acquired data were analysed using Proteus Analysis software (version 8.0.1). All TGA measurements were performed in duplicate (*n* = 2).

#### 2.4.2. Differential Scanning Calorimetry

Differential scanning calorimetry (DSC) measurements were performed using a NEXTA DSC 600 instrument (Hitachi High-Tech, Tokyo, Japan) under a nitrogen atmosphere (50 mL min^−1^). A heating–cooling–heating cycle was applied from 30 to 200 °C at a rate of ± 10 °C min^−1^. The degree of crystallinity (*X_C_*) was calculated according to Equation (1):

(1) $$\chi_{c}\left(\%\right)=\;\frac{{∆H}_{m}{-∆H}_{cc}}{\varphi_{PLA}\times \;{∆H}_{f}^{0}}$$

where ∆*H_m_* is the melting enthalpy, ∆*H_c_*_c_ is the cold crystallization enthalpy, *φ_PLA_* is the mass fraction of PLA in the sample, and ∆*H*^0^*_f_* is the enthalpy of fusion of 100% crystalline PLA, taken as 93 J g^−1^ [43].

#### 2.4.3. Rheology

Oscillatory rheological measurements were performed based on the general principles of ASTM D4440 using a rotational rheometer HR-10 (TA Instruments|Waters, New Castle, DE, USA) equipped with a parallel-plate geometry (25 mm diameter) and a 1.0 mm gap. Complex viscosity (η*), storage modulus (G′), and loss modulus (G″) were obtained from frequency-sweep measurements, with G′ and G″ corresponding to the elastic and viscous components of the complex modulus, respectively. Prior to testing, the samples were vacuum-dried at 80 °C for 4 h. All measurements were conducted at 190 °C under ambient atmosphere. To minimize possible time-dependent degradation during testing, the frequency sweeps were performed from high to low frequency immediately after thermal equilibration, using the same protocol for all formulations. The linear viscoelastic region (LVR) was first determined at 190 °C by strain-amplitude sweeps at 1 Hz, and a strain of 1% (within the LVR) was selected. Oscillatory frequency sweeps were then carried out at 190 °C from 100 to 0.1 Hz using 1% strain. Before each sweep, the samples were equilibrated at the test temperature and excess material was trimmed after gap setting to ensure consistent edge conditions.

#### 2.4.4. Mechanical Testing

Mechanical characterization included impact, tensile, and flexural tests. Impact resistance was evaluated by the Charpy method using the ISO 179-1:2026 [44] specimen geometry, with an INSTRON CEAST 9050 pendulum impact system. A hammer with an impact energy of 2.5 J was used, and all specimens were notched with a 2 mm V-notch prior to testing. The tests were conducted using a support span of 62 mm, and 10 specimens (80 × 10 × 4 mm^3^) were tested for each sample.

Tensile and flexural tests were carried out using the universal testing machine AGX-V 50 kN (Shimadzu Corporation, Kyoto, Japan), controlled by TRAPEZIUMX-V software (version 2.02, Shimadzu Corporation, Kyoto, Japan). Tensile tests were performed according to ASTM D638-22 [45] using a 50 kN load cell and a crosshead speed of 1 mm min^−1^, with dog-bone Type V specimens and a gauge length (GL) of 7.62 mm. Flexural tests were performed according to ISO 178:2019 [46] under a three-point bending configuration using a 5 kN load cell and a crosshead speed of 2 mm min^−1^. The flexural tests were conducted with a support span of 64 mm, and 10 specimens (80 × 10 × 4 mm^3^) were tested for each sample.

For the melt-spun filaments, tensile tests were performed in accordance with ASTM D3822/D3822M-14(2020) [47] using a 100 N load cell, a clamping length of 25 mm and a testing speed of 250 mm min^−1^. Fibre tensile testing was also performed on 10 specimens per sample.

#### 2.4.5. Morphology

Scanning electron microscopy (SEM) analyses were performed using a field-emission scanning electron microscope (FE-SEM), FEI Nova NanoSEM 200 (Hillsboro, OR, USA), operated at an accelerating voltage of 10.0 kV. Fracture-surface micrographs of the injection-moulded composites were obtained from tensile test specimens after mechanical failure. The morphology of the melt-spun monofilaments was also analyzed, considering both their surface and cross-sectional morphology. In this case, filaments were collected from the bobbins and fractured under cryogenic conditions in liquid nitrogen in order to expose the internal fracture surface. The lateral surface of the filaments was also examined. Prior to imaging, all samples were sputter-coated with a thin conductive Au/Pd layer (80/20 wt.%).

#### 2.4.6. Statistical Analysis

Statistical analysis was performed using one-way analysis of variance (ANOVA), followed by Tukey’s HSD post hoc test for multiple comparisons, with the significance level set at *p* < 0.05. For thermal analysis, statistical comparisons were performed among PLA-add and the PLA/cellulose composite formulations, while neat P200 and MCC were included as reference materials and were not considered in the statistical comparison. For mechanical and filament properties, statistical comparisons were performed among the formulations represented in each dataset. Different lowercase letters indicate statistically significant differences between formulations.

## 3. Results and Discussion

### 3.1. PLA/Cellulose Biocomposites

#### 3.1.1. Thermal Analysis

**Figure 2:**
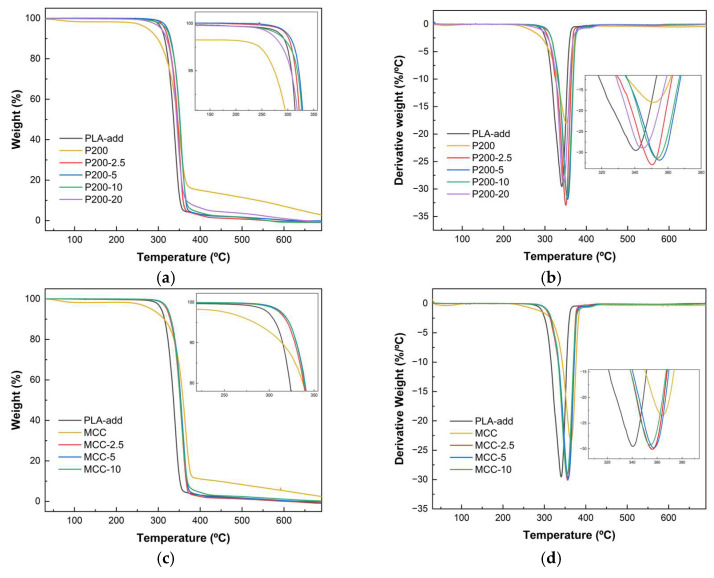
(**a**,**b**) Thermogravimetric (TGA) and derivative thermogravimetric (DTG) curves of PLA-add, neat P200, and PLA/P200 composites containing 2.5, 5, 10, and 20 wt.% cellulose; (**c**,**d**) TGA and DTG curves of PLA-add, neat MCC, and PLA/MCC composites containing 2.5, 5, and 10 wt.% cellulose. Insets show magnified views of the main degradation region.

The thermogravimetric behaviour of the PLA-based formulations is shown in Figure 2, while the main degradation parameters are summarized in Table 2, namely T_5%_, T_onset_, and T_peak_. In addition to PLA-add and the composite formulations, Figure 2 also includes the thermograms of the neat cellulose reinforcements (P200 and MCC), which help to interpret the contribution of the cellulosic phase to the overall degradation behaviour. PLA-add exhibited a typical single-step degradation profile, with T_onset_ = 321.2 °C and T_peak_ = 341.6 °C. The cellulose-filled formulations largely preserved this dominant degradation event, indicating that the matrix remained the main contributor to the TGA response after cellulose incorporation. Similar behaviour has been reported for PLA/cellulose systems in which the degradation of the reinforcement overlaps with that of the matrix, leading to a predominant single degradation region in the composite thermograms [43,48]. The early degradation region should be interpreted separately from the main decomposition step. As shown in Table 2, neat P200 exhibited a lower T_5%_ than PLA-add, indicating an earlier onset of mass loss for the cellulosic reinforcement. This behaviour is consistent with the higher moisture sensitivity of cellulosic fibres and with the onset of cellulose depolymerization occurring at lower temperatures than the main degradation of the more ordered cellulose fraction [43]. Regarding the composite formulations, the incorporation of P200 shifted the main degradation region of PLA-add to higher temperatures at low and intermediate loadings, as shown in Figure 2a,b. The strongest numerical effect was observed for P200-5, which reached T_onset_ = 336.2 °C and T_peak_ = 356.0 °C, corresponding to increases of about 15 °C and 14 °C, respectively, relative to PLA-add. The statistical analysis added to Table 2 showed that all P200-containing composites exhibited T_onset_ values significantly higher than PLA-add; however, the differences among the P200 composites were not statistically significant. Therefore, P200-5 should be interpreted as the numerical maximum of the series rather than as a statistically distinct optimum. This indicates that P200 delayed the onset of the main degradation event, although the effect was more marked at low and intermediate contents. Since TGA does not allow a single degradation mechanism to be assigned, this effect is interpreted here as a modest stabilising contribution of the dispersed cellulosic phase to the PLA-rich degradation region. However, this effect was not monotonic. As shown by the shift in the DTG peak in Figure 2b and the values listed in Table 2, P200-20 exhibited lower T_onset_ and T_peak_ than the lower-loaded P200 composites. Although its T_onset_ remained significantly higher than that of PLA-add, its T_5%_ and T_peak_ values were not significantly different from the PLA-add reference, in contrast with the lower-loaded P200 composites. This indicates that the stabilizing effect became less evident at high fibre loading. This attenuation may be partly related to the larger relative contribution of the cellulosic phase at 20 wt.%, particularly in the early degradation region, as neat P200 exhibited a lower T_5%_ than PLA-add.

The MCC series showed a milder and more stable response over the investigated range. As shown in Figure 2c,d and Table 2, neat MCC exhibited higher T_onset_ and T_peak_ than PLA-add, although its lower T_5%_ again indicates that the early mass-loss region should be interpreted separately from the main degradation step. After incorporation into the matrix, all MCC-filled formulations exhibited T_5%_, T_onset_, and T_peak_ values significantly higher than PLA-add within the investigated range. However, unlike the P200 series, the differences within the MCC group were more limited: no statistically significant differences were observed among MCC-2.5, MCC-5, and MCC-10 for the degradation parameters considered, indicating a comparatively mild stabilizing contribution without a clear monotonic dependence on cellulose content. Similar filler-content-dependent thermal responses have been reported for MCC-containing PLA-based systems, where limited filler additions improved degradation temperatures, whereas further increases reduced the extent of that benefit [49].

These TGA results indicate that cellulose incorporation did not compromise the thermal stability of PLA-add within the investigated range. The main statistically supported effect was the increase in T_onset_ for all PLA/cellulose composites relative to PLA-add, while T_5%_ and T_peak_ were more sensitive to cellulose loading, particularly in the P200 series. This is particularly relevant from a processing standpoint, since all formulations remained thermally stable well above the selected melt-spinning temperature (180–190 °C), indicating that gross thermal instability is unlikely within the chosen processing window.

**Figure 3:**
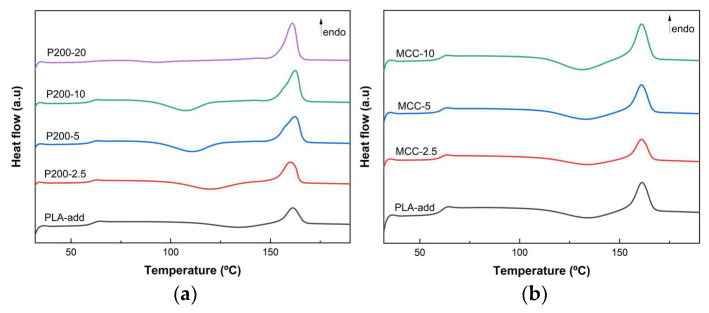
(**a**) Differential scanning calorimetry (DSC) curves of PLA-add and PLA/P200 composites containing 2.5, 5, 10, and 20 wt.% cellulose, obtained during the second heating cycle; (**b**) DSC curves of PLA-add and PLA/MCC composites containing 2.5, 5, and 10 wt.% cellulose, obtained during the second heating cycle.

Whereas TGA mainly evidences the thermal stability of the formulations during heating to degradation, the DSC results reveal how cellulose affects the thermal transitions and, more importantly, the crystallization behaviour of the PLA phase. The calorimetric behavior of the PLA-based formulations is presented in Figure 3, and the main thermal transitions and crystallization-related parameters obtained from the second heating cycle are summarized in Table 3, namely T_g_ (glass transition temperature), T_cc_ (cold crystallization temperature), T_m_ (melting temperature), Δ*H_m_* (melting enthalpy), Δ*H_cc_* (cold crystallization enthalpy), and *X_c_* (degree of crystallinity). Within the investigated composition range, cellulose incorporation had only a limited effect on the fundamental thermal transitions of the PLA-add matrix. T_g_ remained within 59.9–61.5 °C, while T_m_ stayed within 160.8–162.8 °C for all formulations, indicating that cellulose did not markedly alter the segmental mobility of the amorphous PLA phase or the stability of the crystals formed. Similar behaviour has been reported for PLA/cellulose systems, in which the most significant calorimetric changes are usually observed in crystallization-related parameters rather than in T_g_ or T_m_ [43,50].

In contrast, the crystallization behaviour was clearly modified by cellulose addition. A cold-crystallization peak was detected for both PLA-add and the cellulose-reinforced composites; however, the crystallization temperature progressively decreased with increasing cellulose content. This behaviour indicates that cellulose facilitated the crystallization process, allowing the PLA chains to reorganize and crystallize at lower temperatures during reheating. This interpretation is especially relevant for PLA because its crystallization kinetics are intrinsically slow and strongly dependent on the presence of effective nucleating surfaces [43,51]. A marked difference was observed between the P200 and MCC series. In the P200 formulations, T_cc_ decreased substantially with increasing fibre content, from 122.1 °C for P200-2.5 to 95.0 °C for P200-20, indicating that crystallisation occurred more readily in the presence of this reinforcement. This behaviour is consistent with a stronger nucleating effect in the P200 series, likely associated with its fibrous morphology. Similar cellulose-induced changes in PLA crystallization behaviour have been reported for PLA-based multiscale biocomposites and for PLA reinforced with cellulose nanofibers [43,50,52]. The MCC series showed a more moderate response. Although all MCC-containing formulations exhibited a detectable T_cc_, the shift was much smaller, with T_cc_ remaining within a relatively narrow range.

Despite the progressive decrease in T_cc_ observed for the P200 series, the evolution of *X_c_* was decoupled from the T_cc_ shift. For P200-2.5, P200-5, and P200-10, Δ*H_cc_* remained close to Δ*H_m_*, indicating that crystallization occurred predominantly during the second heating cycle and that only a limited crystalline fraction had developed during the preceding cooling step. P200-20 displayed a distinct enthalpy balance: Δ*H_m_* remained comparable to that of P200-10, whereas Δ*H_cc_* decreased sharply from 15.5 to 2.8 J g^−1^. Since X_c_ was calculated from the difference between Δ*H_m_* and Δ*H_cc_* and normalized by the PLA fraction, the high *X_c_* value of P200-20 derives mainly from the reduced residual cold-crystallization contribution during reheating. A similar enthalpy-based relationship between reduced cold-crystallization contribution and increased PLA crystallinity has been reported for PLA/CNC films, although with a more gradual composition dependence than that observed for P200-20 [53]. This behaviour is consistent with more extensive PLA crystallization during the preceding cooling step, promoted by the higher density of fibrous cellulose surfaces at 20 wt.% P200. Thus, the progressive decrease in T_cc_ reflects enhanced nucleation, whereas substantial crystal development before the second heating scan became evident only at the highest P200 loading. In the MCC series, the T_cc_ shift was more limited and *X_c_* increased only moderately within the investigated range, indicating a milder crystallization response. Accordingly, the calculated *X_c_* values describe the crystallization response of the PLA phase under the applied DSC protocol, rather than a direct contribution from cellulose crystallinity or a standalone predictor of the composite mechanical response. The DSC results therefore distinguish the fibrous and particulate reinforcements through the extent to which they affect both nucleation and crystal development of the PLA phase. From a processing perspective, enhanced crystallization during cooling may favour faster structure build-up, whereas a milder crystallization response is generally consistent with a broader thermal-processing window. At the same time, excessively rapid crystallization may reduce the available drawing window during melt spinning [50].

#### 3.1.2. Rheological Behaviour

**Figure 4:**
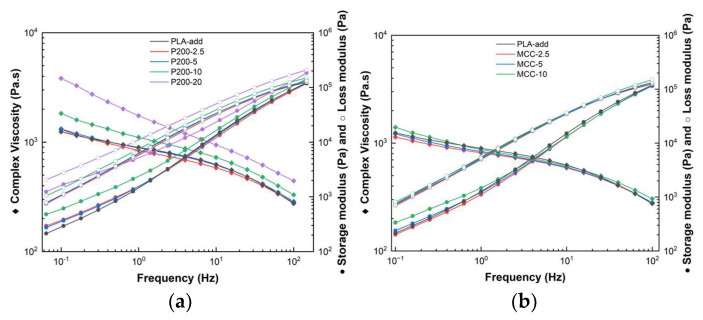
(**a**) Frequency dependence of complex viscosity (η*, diamond symbols), storage modulus (G′, filled circles), and loss modulus (G″, open circles) of PLA-add and PLA/P200 composites containing 2.5, 5, 10, and 20 wt.% cellulose, measured at 190 °C; (**b**) frequency dependence of complex viscosity (η*, diamond symbols), storage modulus (G′, filled circles), and loss modulus (G″, open circles) of PLA-add and PLA/MCC composites containing 2.5, 5, and 10 wt.% cellulose, measured at 190 °C.

Figure 4 compares the frequency dependence of complex viscosity (η*), storage modulus (G′) and loss modulus (G″) for PLA-add and the PLA/cellulose composites reinforced with P200 (Figure 4a) or MCC (Figure 4b) at 190 °C. The curves correspond to mean values from three measurements; the standard deviations remained below the symbol size over the analyzed frequency range and are therefore not visually distinguishable in the plots. Since the compatibilizer and processing aid were kept constant in all formulations, the rheological differences observed in Figure 4 can be mainly attributed to the progressive replacement of PLA by cellulose and to the distinct reinforcement morphology, namely fibrous P200 and particulate MCC. All materials exhibit shear-thinning behaviour, with η* decreasing as frequency increases, while G′ and G″ increase over the investigated frequency range. The G″ curves generally remained above G′, indicating that the formulations retained a predominantly viscous melt response over the analysed frequency window, although the elastic contribution became more evident with cellulose incorporation. This contrast between formulations was more pronounced at low frequency, where filler-related structuring and relaxation processes are more evident, and became less marked at higher frequency, where the response was increasingly governed by the PLA-rich melt matrix [50,54,55,56]. In the MCC series, the rheological response remains close to that of PLA-add up to 10 wt.% cellulose. Only a slight increase in η* is observed at low frequency for MCC-10, while the increase in G′ remains modest; for example, at 0.1 Hz, G′ increases from 214.7 Pa for PLA-add to 336.5 Pa for MCC-10. Similarly, G″ shows only a minor upward shift with increasing MCC content, remaining close to the PLA-add response throughout the frequency range. This indicates that, within the investigated composition range, MCC acts predominantly as a particulate filler with limited ability to generate a connected melt structure. This interpretation is consistent with Incarnato et al., who reported that MCC additions up to 12 wt.% do not significantly alter the viscoelastic flow behaviour of PLA [57], and with Shumigin et al., who showed that the rheological effect of cellulose depends not only on its content but also on filler geometry and dispersion state [54].

The P200 series exhibits a markedly different response. While PLA-add, P200-2.5 and P200-5 remain relatively close in η* over most of the frequency range, P200-10 and especially P200-20 display a clear upward shift, most pronounced at low frequency. The same trend is more evident in G′, where P200-20 reaches 1243.7 Pa at 0.1 Hz, corresponding to an almost six-fold increase relative to PLA-add. G″ also increased with P200 loading, particularly for P200-10 and P200-20, consistent with a stronger contribution of the fibrous reinforcement to the overall viscoelastic response of the melt. This behaviour indicates a stronger contribution of low-frequency structuring, consistent with the higher effective aspect ratio of P200 and the resulting increase in fibre–fibre contacts and transient connectivity. Similar low-frequency reinforcement has been reported for PLA/cellulose systems when filler morphology favours network formation or inter-filler connectivity [50,55,56]. Although the present response is less pronounced than that typically reported for CNC- or CNF-filled PLA, which is expected given the larger size and lower specific surface area of P200, the same mechanistic tendency is evident.

Increasing cellulose loading necessarily reduces the PLA matrix fraction and its chain entanglement contribution; therefore, the fact that η*, G′ and G″ still increase with loading indicates that the reinforcing contribution of the cellulose phase more than compensates for the reduced matrix contribution. Although G″ remained above G′ over the investigated frequency range, G′ was measurable for all formulations and increased with cellulose incorporation, particularly in the P200 series. This indicates that the melts retained an elastic contribution, while maintaining a predominantly viscous response under small-amplitude oscillatory shear. Therefore, the absence of a G′/G″ crossover within the analyzed frequency window should not be interpreted as a lack of melt elasticity or as precluding filament formation under the transient extensional deformation imposed during spinning. The reinforcement-induced increase in viscoelastic response remained limited for MCC up to 10 wt.%, whereas it became more pronounced for P200 at 10 and 20 wt.%, where the fibre morphology promoted stronger low-frequency structuring. Overall, the results show that cellulose wt.% alone is not sufficient to predict melt response. At comparable loadings, particulate MCC produces only minor changes in the viscoelastic response of PLA-add, whereas fibrous P200 generates a stronger increase in low-frequency elasticity and viscosity. From a processing standpoint, MCC-containing formulations are expected to preserve a wider flow window, whereas P200-rich systems provide stronger melt structuring, albeit at the expense of a narrower processing latitude as fibre loading increases.

#### 3.1.3. Mechanical Properties

##### Tensile Properties

**Figure 5:**
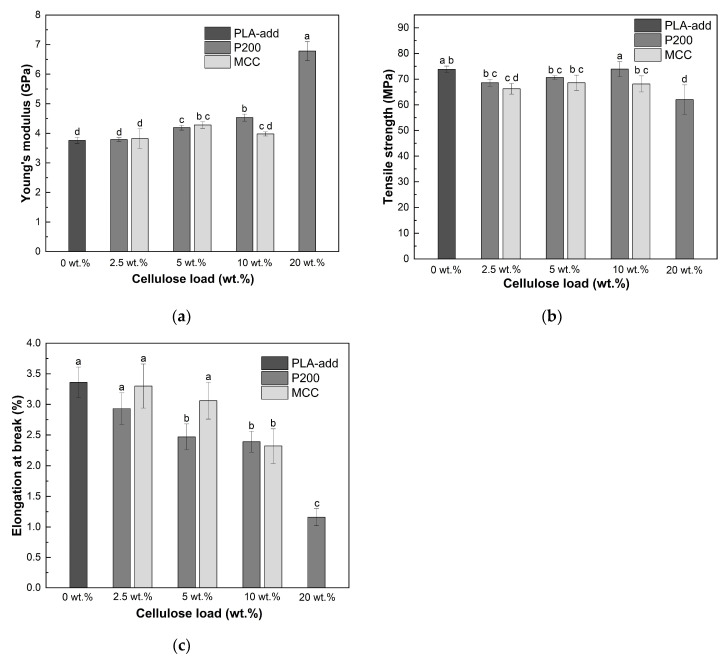
Tensile properties of PLA-add and PLA/cellulose composites containing P200 or MCC at different cellulose loadings: (**a**) Young’s modulus; (**b**) tensile strength; and (**c**) elongation at break. Bars represent mean values and error bars indicate standard deviation. Different lowercase letters indicate statistically significant differences between formulations according to one-way ANOVA followed by Tukey’s HSD test (*p* < 0.05).

Figure 5 shows the tensile response of PLA-add and the corresponding cellulose-filled composites in terms of Young’s modulus, tensile strength, and elongation at break for the P200 and MCC series. Since the compatibilizer/processing-aid package was fixed, the mechanical trends are discussed primarily as a function of cellulose type and loading. Cellulose incorporation mainly affected the tensile response by increasing stiffness and reducing ductility, whereas its effect on tensile strength was more limited. Young’s modulus increased most clearly in the P200 series, with the statistical groupings showing a progressive stiffening effect as fibre loading increased. This response is consistent with the incorporation of rigid cellulosic domains into the PLA matrix and with the influence of reinforcement morphology on load-bearing stiffness. The stiffening effect became particularly evident at high loading, where P200-20 formed the highest statistical group, reaching 6.78 ± 0.32 GPa compared with 3.76 ± 0.10 GPa for PLA-add. In the MCC series, the increase was more moderate and less monotonic, indicating a weaker stiffening effect within the investigated range. This trend is consistent with previous studies showing that more fibrous cellulose reinforcements can produce a stronger stiffening effect than MCC-type particulate fillers in PLA-based systems [28,58,59].

Compared with Young’s modulus, tensile strength varied within a narrower range and showed a less systematic dependence on cellulose loading. Several formulations remained statistically close to PLA-add, whereas the lowest value was observed for P200-20. Therefore, in the present system, the increase in stiffness did not translate into a proportional increase in strength. Accordingly, the tensile-strength response appears to depend not only on reinforcement stiffness, but also on the effectiveness of stress transfer across the PLA/cellulose interface and on the sensitivity to local defects during failure. A similar non-parallel evolution of modulus and tensile strength has been reported for PLA/MCC composites, in which modulus increased slightly while tensile strength decreased or remained nearly unchanged as MCC content increased [58]. In the same way, previous work on PLA composites reinforced with micronized pulp fibres showed that stiffness is more consistently improved than elongation-related properties, whereas the strength response depends more strongly on the specific reinforcement condition and formulation [28].

The reduction in elongation at break was more clearly expressed than the changes in tensile strength. In the P200 series, the decrease became statistically evident from 5 wt.% onwards and reached the lowest value at P200-20. In the MCC series, the loss of ductility was milder, with MCC-2.5 and MCC-5 remaining statistically comparable to PLA-add and a significant reduction becoming evident only at 10 wt.%. Such loss of ductility is commonly associated with the incorporation of rigid cellulosic domains, which constrain matrix deformation and increase the sensitivity of the material to local interfacial defects during tensile loading [28,58]. In the present case, the tensile response therefore differentiates the two cellulose grades clearly: P200 produced the largest stiffness increase and the more evident loss of ductility, whereas MCC led to a more moderate mechanical change over the investigated range.

##### Flexural Properties

**Figure 6:**
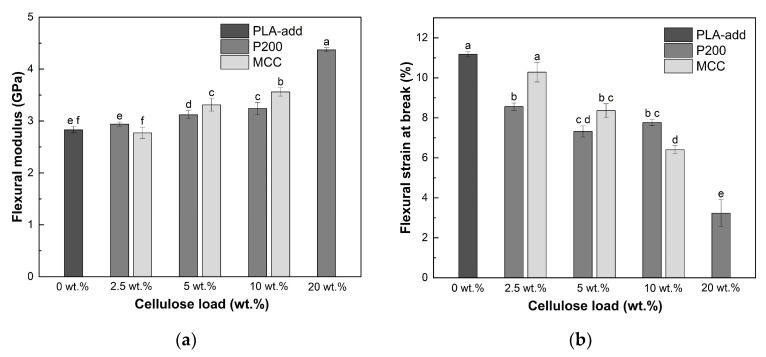
Flexural properties of PLA-add and PLA/cellulose composites containing P200 or MCC at different cellulose loadings: (**a**) flexural modulus; and (**b**) flexural strain at break. Bars represent mean values and error bars indicate standard deviation. Different lowercase letters indicate statistically significant differences between formulations according to one-way ANOVA followed by Tukey’s HSD test (*p* < 0.05).

Figure 6 shows the flexural response of PLA-add and the corresponding cellulose-filled composites in terms of flexural modulus and strain at break for the P200 and MCC series. As in tension, cellulose incorporation increased stiffness under bending and reduced the deformation at break. The flexural modulus increased in both series, with the statistical groupings showing only limited changes at 2.5 wt.% and clearer increases at higher loadings. This behaviour agrees with previous studies on PLA/cellulose composites, in which the addition of cellulose generally increases the flexural modulus because the rigid cellulosic phase contributes more strongly to the overall bending stiffness of the material [28,60,61].

A difference between the two cellulose grades nevertheless emerged. Up to 10 wt.%, the MCC formulations showed flexural modulus values comparable to, and in the present case slightly higher than, those of the corresponding P200 composites. This indicates that under bending, the particulate cellulose phase was already effective in increasing rigidity at low and intermediate loading. Such behaviour is consistent with previous work on PLA/MCC systems, in which all MCC-filled formulations exhibited flexural modulus values above that of neat PLA, with the best response obtained at intermediate loading before a slight decline at higher contents [61]. Similar increases in flexural modulus have also been reported for PLA reinforced with micronized pulp fibres and with wood fibres processed by extrusion and injection moulding [28,61].

The P200 series became clearly distinct only at the highest loading. While its flexural modulus increased more gradually up to 10 wt.%, P200-20 exhibited the highest modulus of the whole set, showing that the fibrous reinforcement produced the strongest stiffening effect once its content became sufficiently high. This observation is in line with previous studies reporting substantial increases in flexural modulus for PLA reinforced with cellulosic fibres, while also indicating that the magnitude of this response depends on fibre characteristics and processing conditions [28,61].

The strain at break decreased in both series, confirming a statistically supported loss of flexibility under bending, although MCC-2.5 remained comparable to PLA-add. For P200, this decrease was particularly marked at 20 wt.%, where the material became clearly less deformable than PLA-add. In the MCC series, the reduction was more moderate up to 5 wt.%, and became more evident at 10 wt.%. This trend is consistent with previous reports showing that higher cellulose contents reduce the deformation at failure of PLA-based composites under bending, while the increase in stiffness is accompanied by lower flexibility [28]. In the present results, the bending response of the two series remained differentiated: MCC provided a more moderate flexural evolution over the investigated range, whereas P200 produced the strongest stiffening effect only at the highest loading, together with the sharpest reduction in strain at break.

##### Impact Properties

**Figure 7:**
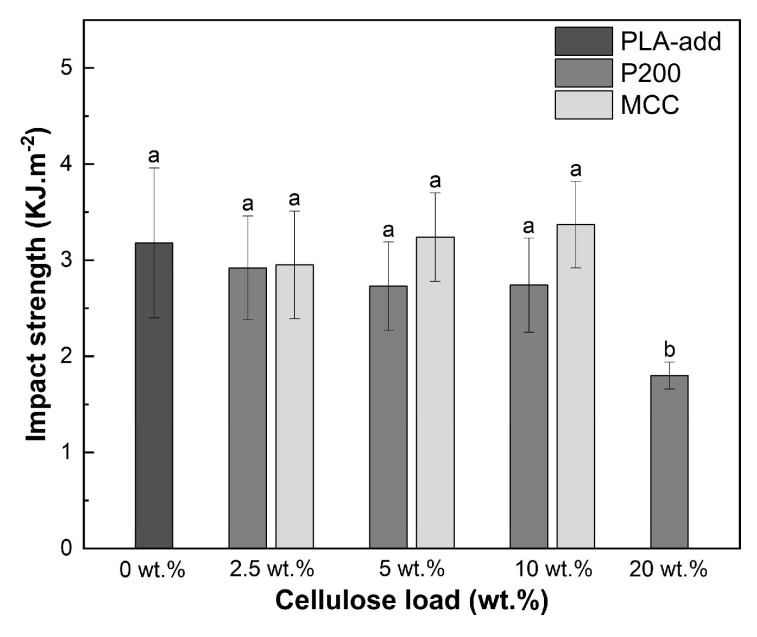
Notched impact strength of PLA-add and PLA/cellulose composites containing P200 or MCC as a function of cellulose loading. Bars represent mean values and error bars indicate standard deviation. Different lowercase letters indicate statistically significant differences between formulations according to one-way ANOVA followed by Tukey’s HSD test (*p* < 0.05).

The notched impact strength of PLA-add and of the corresponding cellulose-filled composites in the P200 and MCC series is presented in Figure 7. PLA-add exhibited an impact strength of 3.2 ± 0.8 kJ·m^−2^. In the P200 series, impact strength showed a numerical decrease with increasing fibre content, remaining statistically comparable to PLA-add up to 10 wt.% and becoming significantly lower only at 20 wt.%, where the value reached 1.8 ± 0.1 kJ·m^−2^. In contrast, all MCC formulations remained statistically comparable to PLA-add over the investigated range, despite the slightly higher mean values observed at 5 and 10 wt.%, with a maximum of 3.4 ± 0.5 kJ·m^−2^ at 10 wt.%. The statistically significant decrease observed for P200-20 indicates that the adverse effect of the fibrous reinforcement on notched impact performance became relevant mainly at the highest loading.

This trend is consistent with the tensile and flexural results, where the P200 series also showed the strongest loss of deformability as cellulose content increased. A reduction in impact performance after cellulose addition has likewise been reported for PLA-based green composites reinforced with micronized pulp fibres, for which the incorporation of cellulose lowered the amount of impact energy absorbed by the material. In notched PLA composites reinforced with bleached kraft softwood pulp, Oliver-Ortega et al. reported that impact strength remained nearly unchanged with increasing fibre content, with values close to 3.1–3.3 kJ·m^−2^, indicating that notched impact can remain relatively stable in some cellulose-reinforced PLA systems. Compared with that behaviour, the decrease observed for P200-20 in the present work is clearly more pronounced [28,62].

The MCC series exhibited a milder response. Up to 10 wt.%, the notched impact strength remained comparable to, or slightly above, that of PLA-add, indicating that this particulate reinforcement did not penalize impact resistance to the same extent as P200 within the investigated range. This more moderate behaviour is consistent with the fact that the MCC series also showed smaller changes in tensile and flexural ductility than the P200 composites. The literature shows that the effect of cellulose on PLA impact behaviour is not unique: Gao and Qiang reported that ball-milled cellulose increased the impact strength of PLA, with higher values obtained at smaller particle size and higher filler content, whereas micronized pulp fibres were reported to reduce the impact strength of PLA-based composites. In this context, the present MCC results can be regarded as an intermediate response, since impact resistance was not substantially improved, but it was also not reduced as severely as in the high-loading P200 formulation [62,63].

#### 3.1.4. Fracture Morphology

**Figure 8:**
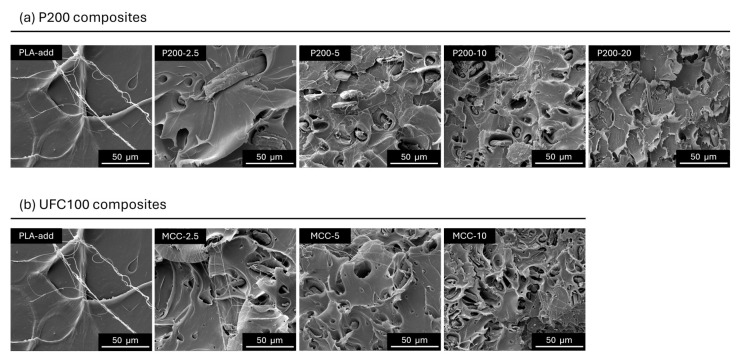
SEM micrographs of the tensile fracture surfaces of PLA-add and PLA/cellulose composites containing (**a**) P200 and (**b**) MCC at different cellulose contents. Scale bar: 50 μm.

Scanning electron micrographs of the tensile fracture surfaces shown in Figure 8 reveal clear morphological differences between PLA-add and the cellulose-filled composites, highlighting the influence of reinforcement geometry, dispersion, and interfacial adhesion on the fracture process. In Figure 8, PLA-add exhibits a relatively smooth and compact fracture surface, with broad cleavage-like regions and little evidence of plastic deformation, which is consistent with the brittle fracture behaviour typically observed for neat PLA [63].

The P200 series shows a markedly different morphology. Even at low fibre loading, the fracture surfaces become rougher and more heterogeneous, and elongated pull-out cavities together with partially extracted fibres are clearly visible in Figure 8a. As cellulose content increases, these features become more frequent and more pronounced, and the fracture surface evolves towards a more irregular fibre-rich morphology with larger voids and more severe matrix tearing, particularly for P200-10 and P200-20. In PLA/cellulose composites, features of this type are commonly associated with incomplete fibre–matrix bonding, since fibre pull-out and interfacial gaps indicate that failure propagates preferentially along the interface rather than through efficient stress transfer to the reinforcement [28,59]. At the same time, the P200 micrographs do not indicate severe agglomeration at low and intermediate loading, suggesting that melt compounding enabled a reasonably effective dispersion of the micronized fibres in the PLA matrix. This interpretation is consistent with previous observations for PLA reinforced with micronized pulp fibres, in which no visible agglomerates or bundles were detected and the fibres remained relatively well distributed in the matrix [28]. In that same study, increasing fibre size and aspect ratio was associated with longer pull-outs and more voids, whereas shorter micronized fibres showed improved embedment in the matrix [28]. The sequence shown in Figure 8a follows the same general tendency: the reinforcement remains incorporated into the matrix, but fibre-related defects become more severe as loading increases. This morphological evolution is consistent with the mechanical response discussed above. The strong increase in tensile and flexural modulus observed for the P200 series indicates that the fibrous phase effectively stiffened the matrix, whereas the simultaneous reduction in elongation at break and the marked drop in notched impact strength at 20 wt.% indicate greater sensitivity to crack initiation and unstable fracture. In this context, the larger pull-out cavities, interfacial voids, and stronger matrix tearing observed in the P200 composites in Figure 8a are consistent with the combination of efficient stiffening and reduced damage tolerance at high loading.

The MCC series exhibits a distinct fracture pattern. Here, the fracture surfaces are also rougher than that of PLA-add, but the dominant features are shorter cavities, particle-sized voids, and localized pull-out sites rather than elongated fibre traces, as shown in Figure 8b. This shorter characteristic defect size is consistent with the particulate nature of the reinforcement. Similar behaviour has been reported for PLA/MCC systems, in which MCC particles were observed to debond and pull out from the PLA matrix in the absence of stronger interfacial interaction [60]. In the present MCC composites, increasing cellulose content leads to a higher density of microvoids and a more irregular fractured matrix, particularly at 10 wt.%, but the fracture surfaces remain less disrupted than in the corresponding P200 formulations. This agrees with the mechanical results, where MCC produced more moderate increases in stiffness and a less severe reduction in ductility and impact resistance within the investigated range. Accordingly, the fracture morphology observed in Figure 8 indicates that the mechanical response depended not only on cellulose content, but also on the way in which reinforcement geometry influenced interfacial failure and defect development during fracture.

### 3.2. Monofilament Production by Melt Spinning

The composite-level characterization presented in Section 3.1 was used as a screening step for the selection of formulations for spinning trials and to examine the transition from moulded-compound behaviour to filament-processing response. Based on this analysis, the 5 wt.% formulations were selected for melt spinning, as they provided the best compromise between thermal stability, rheological processability, mechanical reinforcement, and morphological integrity. At this composition, both cellulose types still preserved the increase in degradation temperatures observed by TGA, while rheological penalties remained moderate and the more pronounced mechanical deterioration observed at higher P200 loadings had not yet become critical. Accordingly, P200-5 and MCC-5 were selected for spinning trials, while PLA-add was included as a reference material and baseline system. In this design, the filament-level comparison is restricted to the selected 5 wt.% formulations and to the processing conditions adopted in this work; it is therefore not intended as a full compositional mapping or formulation-specific optimization of the P200 and MCC series.

#### 3.2.1. Spinnability

The spinnability of the selected formulations was assessed to determine how cellulose morphology influences filament formation, process stability, and the drawable window during melt spinning. Based on the results presented in Section 3.1, P200-5 was selected as the first formulation to be processed, since it was expected to show the highest processing sensitivity due to its stronger melt structuring, higher stiffness, and lower ductility relative to the reference matrix. This expectation was also consistent with the stronger low-frequency viscoelastic response observed for the P200 series and with its more pronounced crystallization tendency relative to MCC, both of which are indicative of a formulation less tolerant to stable stretching during spinning and subsequent drawing. The minimum set of processing conditions required to obtain stable continuous filaments from this formulation was then used as a baseline for PLA-add and MCC-5, enabling a direct comparison among the three systems. For each formulation, two spinning conditions were investigated: a reference condition (Ref), used for direct comparison among formulations, and a limiting draw condition (Lim), corresponding to the highest stable draw condition achieved without filament breakage under the fixed comparative protocol used in this study. In addition to improving filament orientation, the application of the limiting condition made it possible to evaluate the extent to which filament fineness, expressed in terms of linear density and diameter, could be further reduced during processing.

A first practical requirement during spinning was to ensure sufficient melt strength and stable take-up for continuous filament formation. This was achieved by operating at relatively low melt temperatures, between 180 and 190 °C, and at a constant throughput of 14 g min^−1^. Under these conditions, stable continuous monofilaments could be produced for all three formulations, although their processing behaviour differed markedly (Table 4). Under the reference condition, PLA-add exhibited the most stable response, yielding the most uniform filaments, with a linear density of about 860 dtex and a diameter of 274 µm. MCC-5 behaved similarly, showing only a slight increase in diameter and maintaining adequate flexibility for stable processing. In contrast, P200-5 exhibited a clearly less favourable behaviour. The resulting filaments were stiffer, displayed a rougher surface, and showed poorer handling along the godets, which negatively affected process stability. In addition, the larger filament diameter obtained under the same nominal draw conditions suggests a lower effective deformation during spinning and is consistent with a less homogeneous filament structure [12,64]. This interpretation is also consistent with the filament morphology discussed later, where rougher surfaces and internal heterogeneity are observed in the cellulose-containing filaments.

The differences among formulations became more evident under the limiting draw condition. PLA-add displayed the widest drawable window, with the melt draw ratio increasing from 20.6 to 132.5 and the draw ratio from 1.1 to 1.3, resulting in a marked reduction in both linear density and filament diameter. This behaviour indicates that the reference PLA-based system could sustain a substantially higher level of deformation during processing, thereby allowing more effective filament stretching and orientation development [12,64]. By contrast, both cellulose-containing formulations showed a much more restricted increase in drawability. For both P200-5 and MCC-5, the melt draw ratio increased only to 41.2, while the draw ratio remained unchanged at 1.1. Accordingly, the reduction in linear density and diameter was much less pronounced than for PLA-add, indicating lower draw efficiency and a more limited degree of structural refinement during spinning. These results suggest that cellulose incorporation reduced the deformation capacity of the filaments under the present conditions and limited the extent to which additional orientation could be imposed during processing [33]. Within the fixed comparative spinning protocol adopted in this study, the limiting draw condition represents the highest stable draw level achieved under the selected processing conditions, rather than an intrinsic drawability limit of the cellulose-containing formulations.

The reduced drawable window of the cellulose-filled systems is consistent with the composite-level results presented in Section 3.1. In those materials, cellulose incorporation increased stiffness and reduced ductility, with a more pronounced effect for P200. The stronger limitation observed for P200-5 is therefore consistent with the combined effects of reinforcement morphology, reduced filament flexibility, stronger low-frequency melt structuring, and less favourable structural development during spinning. The available literature on melt-spun PLA/cellulose fibres similarly indicates that cellulose morphology, dispersion quality, and interfacial compatibility are key factors governing drawability and final fibre performance [33,34]. In this context, the more severe limitation observed for P200-5 may also reflect greater flow perturbation and structural heterogeneity during stretching, associated with its more fibrous reinforcement geometry. MCC-5 also reduced stretchability relative to PLA-add, but to a lesser extent, indicating that the more particulate morphology was less detrimental to spinning stability under the present conditions.

Comparison of the three formulations highlights the influence of cellulose morphology on the accessible processing window within the selected 5 wt.% formulations and under the adopted processing conditions. PLA-add showed the highest drawability and the most stable spinning response, whereas MCC-5 retained a comparatively more favourable balance between reinforcement and processability. By contrast, P200-5 was more strongly limited under the present melt-spinning conditions, indicating a less favourable response to filament formation and drawing.

#### 3.2.2. Mechanical Properties

**Figure 9:**
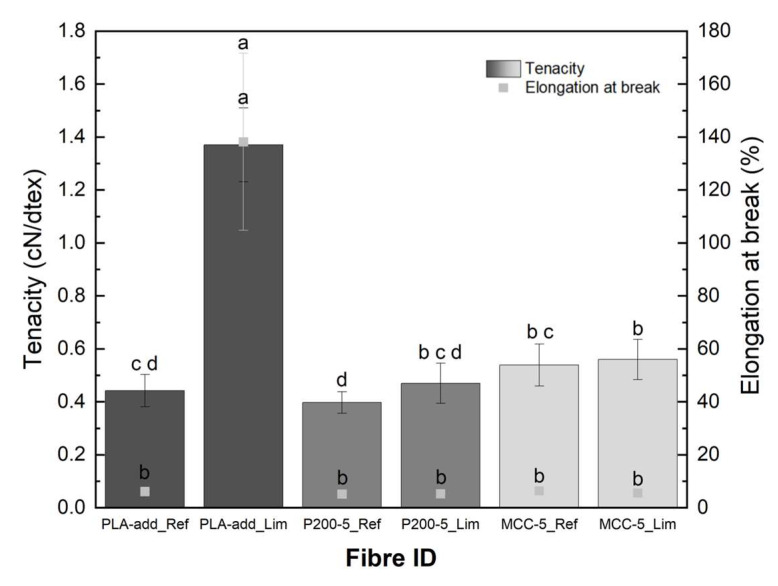
Tenacity and elongation at break of melt-spun PLA-add, P200-5, and MCC-5 monofilaments produced under the reference (Ref) and limiting (Lim) draw conditions. Bars and square symbols represent mean values, and error bars indicate standard deviation. Statistical comparisons were performed separately for tenacity and elongation at break; different lowercase letters indicate statistically significant differences within each property according to one-way ANOVA followed by Tukey’s HSD test (*p* < 0.05).

The mechanical performance of the melt-spun monofilaments, in terms of tenacity and elongation at break, is presented in Figure 9. A clear effect of the applied drawing condition is observed, particularly for PLA-add. The statistical comparisons, performed separately for tenacity and elongation at break, confirm that PLA-add_Lim formed the highest tenacity group and was the only condition with a statistically distinct increase in elongation at break. Under the limiting draw condition, PLA-add showed a pronounced increase in tenacity, reaching about 1.37 cN/dtex, together with a substantial increase in elongation at break compared with the reference condition. This behaviour is consistent with the broader drawable window identified in Section 3.2.1 and indicates that the PLA-based reference system was able to sustain a much higher degree of stretching and orientation development during processing. Similar behaviour has been reported for melt-spun PLA systems, in which drawing strongly affects mechanical performance and may lead not only to increases in strength, but also to higher elongation at break when the oriented structure develops under suitable drawing conditions [65,66].

By contrast, the cellulose-containing filaments showed a much weaker mechanical response to the limiting draw condition. For both P200-5 and MCC-5, the increase in tenacity was relatively modest, and elongation at break remained low compared with PLA-add. This suggests that the cellulose-filled filaments were much less able to convert the additional drawing into effective mechanical property development. This behaviour is consistent with the more limited drawability observed for the biocomposite systems during spinning, which constrained the extent of deformation and thus reduced the degree of additional orientation achieved during processing [65]. It is also in line with the fracture features observed at composite level in Section 3.1.4, where the P200 series showed more pronounced fibre-related cavities and interfacial defects, whereas the MCC series was characterized by shorter particle-scale heterogeneities and localized debonding features.

When the three formulations are compared under the reference condition, the differences are less pronounced. PLA-add, P200-5, and MCC-5 all exhibited relatively low tenacity values, indicating that under this low-draw condition the degree of structural development remained limited in all cases. Even so, MCC-5 showed a higher mean tenacity than P200-5 under the reference condition, while P200-5 exhibited the least favourable response. Under the limiting draw condition, the two cellulose-containing filaments remained in lower statistical groups than PLA-add_Lim, and their differences were much less pronounced than the gap relative to the PLA-add limiting condition.

The contrast becomes clearer when elongation at break is considered. The marked increase observed for PLA-add under the limiting draw condition suggests that the drawn PLA filament developed a more stable deformation response, whereas the cellulose-filled systems remained comparatively fragile. This interpretation is consistent with previous studies showing that low draw ratios in melt-spun PLA yarns are associated with brittle behaviour, while higher draw ratios promote a more favourable mechanical response through improved molecular orientation [66]. In the present case, however, the beneficial effect of increased drawing was largely suppressed by cellulose incorporation, especially for P200-5. The slightly more favourable mean response of MCC-5 relative to P200-5 is consistent with the spinnability results discussed above, where the more particulate morphology was less disruptive to stable filament formation and drawing than the fibrous reinforcement.

#### 3.2.3. Filament Morphology

**Figure 10:**
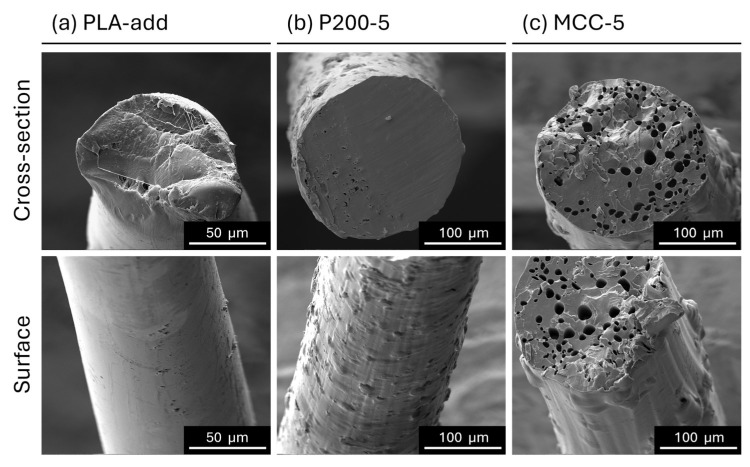
SEM images of PLA-add, P200-5, and MCC-5 monofilaments produced under the limiting draw condition. Cryo-fractured cross-sectional views are shown in the top row, and representative lateral/oblique surface views are shown in the bottom row. Scale bars: 50 µm for PLA-add images and 100 µm for P200-5 and MCC-5 images.

The morphology of the monofilaments produced under the limiting draw condition was examined by SEM, and representative cryo-fractured cross-sectional and lateral/oblique surface images are shown in Figure 10. Under this processing condition, the final filament fineness differed markedly among formulations, with PLA-add reaching a substantially lower diameter than the cellulose-containing systems, as shown in Table 4. Therefore, the SEM images are interpreted here as representative morphological views of each filament rather than as a basis for quantitative comparison of void size or void density. PLA-add exhibited the most uniform morphology, with a comparatively smooth surface and a compact fracture section. This observation is consistent with its better spinnability and with the stronger mechanical improvement obtained under the limiting draw condition.

Both cellulose-containing systems showed clear morphological heterogeneity, although in different ways. P200-5 exhibited the most pronounced surface irregularity, with visible longitudinal roughness and surface perturbations along the filament. This feature is consistent with the poorer handling and reduced drawability observed during spinning and suggests that the more fibrous reinforcement morphology disturbed the external geometry of the filament more strongly during processing. A similar tendency has been reported for melt-spun PLA/cellulose systems, in which cellulose incorporation increased surface roughness, promoted aggregation, and restricted fibre drawability [35].

MCC-5, in turn, exhibited visible internal heterogeneity in the cryo-fractured section, with cavities and discontinuities distributed across the fractured surface. These features are consistent with local interfacial discontinuities, filler-rich regions, and/or pull-out around heterogeneous domains. This interpretation is in agreement with previous reports identifying MCC as less suitable than nanometric cellulose for PLA melt spinning, owing to its more restricted drawability and poorer dispersion within the PLA matrix [33].

The two cellulose-containing systems differed mainly in the dominant form of morphological heterogeneity. P200-5 was characterized primarily by surface irregularity, whereas MCC-5 showed more evident internal discontinuities in the fractured section. In P200-5, the rougher and more disturbed filament surface is consistent with the reduced process stability observed during spinning and drawing, while in MCC-5 the internal discontinuities are consistent with the limited mechanical improvement obtained under the limiting draw condition. This interpretation is also consistent with the fracture morphology observed for the injection-moulded composites in Section 3.1.4, where the P200 series showed more elongated fibre-related defects, whereas the MCC series was characterized by shorter particle-scale cavities and localized debonding features. In turn, this difference is reflected in the overall behaviour of the two systems, with MCC-5 retaining a slightly more favourable spinning response than P200-5, yet still showing only limited mechanical improvement under the limiting draw condition.

## 4. Conclusions

This study showed that cellulose morphology influences the processability and performance of PLA-based biocomposites for monofilament production by melt spinning. At composite level, both cellulose types shifted the main degradation region of PLA-add to higher temperatures, with the increase in T_onset_ representing the main statistically supported thermal effect, while their effect on rheological and mechanical behaviour depended markedly on reinforcement morphology. P200 promoted a stronger increase in low-frequency melt structuring, stiffness, and crystallization tendency, but also a more pronounced reduction in ductility; the decrease in notched impact resistance became significant only at the highest P200 loading. MCC produced a more moderate overall response, indicating a less severe perturbation of the PLA matrix. These differences were also reflected in fracture morphology, with the P200 series showing more elongated fibre-related defects and the MCC series exhibiting shorter particle-scale cavities and localized debonding features.

Based on this balance of properties, P200-5 and MCC-5 were selected for melt spinning. Stable continuous monofilaments were obtained for both cellulose-containing systems, confirming the feasibility of processing the selected formulations by melt spinning under the adopted conditions. However, neither P200-5 nor MCC-5 matched the drawable window or mechanical development of PLA-add. PLA-add showed the widest drawable window and the strongest mechanical improvement under increased drawing. Among the cellulose-containing systems, MCC-5 retained a more favourable balance between reinforcement and processability, whereas P200-5 imposed a stronger limitation on filament formation, drawable range, and mechanical development. SEM observations supported this interpretation, showing that P200-5 was mainly associated with surface irregularity, while MCC-5 exhibited visible internal discontinuities in the fractured section. The filament-level conclusions are therefore restricted to the selected 5 wt.% formulations and to the processing conditions adopted in this work. Further work should therefore focus on formulation-specific optimization of the melt-spinning process, including temperature profile, quenching conditions, drawing schedule, compatibilizer level, and processing-aid content, in order to distinguish intrinsic material limitations from process-related constraints in cellulose-containing PLA monofilaments.

## Figures and Tables

**Table 1:** Formulations of PLA with additives (PLA-add) and PLA/cellulose composites reinforced with P200 or MCC (wt.% of total formulation).

Sample ID	Cellulose Type	Cellulose (wt.%)	PLA (wt.%)	Compatibilizer (wt.%)	Processing Aid (wt.%)
PLA-add	-	0	90	5	5
P200-2.5	P200	2.5	87.5	5	5
P200-5	P200	5	85	5	5
P200-10	P200	10	80	5	5
P200-20	P200	20	70	5	5
MCC-2.5	MCC	2.5	87.5	5	5
MCC-5	MCC	5	85	5	5
MCC-10	MCC	10	80	5	5

**Table 2:** Thermogravimetric degradation parameters of PLA-add, neat cellulose reinforcements (P200 and MCC), and PLA/cellulose composites containing P200 or MCC. T_5%_, temperature at 5% weight loss; T_onset_, onset degradation temperature; T_peak_, temperature at the maximum degradation rate.

Sample	T_5%_ (°C)	T_onset_ (°C)	T_peak_ (°C)
PLA-add	307.5 ± 0.7 ^c^	321.2 ± 0.0 ^b^	341.6 ± 0.2 ^c^
P200	276.0 ± 2.0	319.9 ± 0.2	351.6 ± 0.4
P200-2.5	318.5 ± 5.0 ^ab^	335.0 ± 5.0 ^a^	354.9 ± 6.4 ^ab^
P200-5	320.5 ± 0.7 ^a^	336.2 ± 0.8 ^a^	356.0 ± 1.6 ^ab^
P200-10	318.0 ± 0.0 ^ab^	334.8 ± 1.2 ^a^	354.0 ± 0.4 ^ab^
P200-20	310.0 ± 0.0 ^bc^	330.5 ± 1.1 ^a^	348.1 ± 0.9 ^bc^
MCC	281.5 ± 1.5	338.9 ± 0.3	363.5 ± 0.8
MCC-2.5	321.5 ± 2.1 ^a^	337.1 ± 1.8 ^a^	358.3 ± 1.3 ^a^
MCC-5	320.5 ± 2.1 ^a^	336.1 ± 0.9 ^a^	356.8 ± 0.8 ^ab^
MCC-10	320.0 ± 2.8 ^a^	334.8 ± 0.9 ^a^	354.8 ± 0.2 ^ab^

Values are reported as mean ± standard deviation from duplicate measurements (*n* = 2). Different superscript letters within the same column indicate statistically significant differences among PLA-add and PLA/cellulose composite formulations according to one-way ANOVA followed by Tukey’s HSD test (*p* < 0.05). Neat P200 and MCC were included as reference materials and were not considered in the statistical comparison.

**Table 3:** Thermal transitions and crystallization-related parameters of PLA/cellulose composites obtained from the second heating cycle. T_g_, glass transition temperature; T_cc_, cold crystallization temperature; T_m_, melting temperature; ∆*H_m_*, melting enthalpy; ∆*H_cc_*, cold crystallization enthalpy; *X_c_*, degree of crystallinity.

Sample	T_g_ (°C)	T_cc_ (°C)	T_m_ (°C)	∆*H_m_* (J g^−1^)	∆*H_cc_* (J g^−1^)	*X_c_* (%)
PLA-add	61.2 ± 0.0	134.7 ± 0.2	161.5 ± 0.2	9.5 ± 1.0	6.3 ± 0.8	3.2 ± 0.6
P200-2.5	60.9 ± 0.1	122.1 ± 1.2	160.8 ± 0.2	21.8 ± 1.0	20.3 ± 0.1	1.3 ± 0.6
P200-5	61.2 ± 0.0	111.9 ± 0.2	162.8 ± 0.1	21.5 ± 0.9	18.7 ± 0.1	2.9 ± 0.6
P200-10	60.8 ± 0.1	108.2 ± 0.1	162.3 ± 0.2	19.7 ± 0.9	15.5 ± 0.1	5.1 ± 0.6
P200-20	59.9 ± 0.5	95.0 ± 0.6	161.2 ± 0.3	19.9 ± 1.5	2.8 ± 0.3	25.1 ± 1.1
MCC-2.5	61.5 ± 0.1	134.8 ± 0.2	161.7 ± 0.3	11.1 ± 1.4	7.2 ± 0.6	3.9 ± 0.9
MCC-5	61.3 ± 0.1	133.4 ± 0.1	161.4 ± 0.4	14.8 ± 1.1	10.3 ± 1.0	5.0 ± 0.7
MCC-10	61.4 ± 0.3	131.9 ± 0.2	161.6 ± 0.5	18.0 ± 0.2	13.8 ± 0.2	5.7 ± 0.1

**Table 4:** Melt-spinning conditions and resulting filament fineness characteristics of PLA-add, P200-5, and MCC-5 monofilaments under reference (Ref) and limiting (Lim) draw conditions, obtained at a constant throughput of 14 g min^−1^.

Fibre ID	MDR	DR	Linear Density (dtex)	Diameter (µm)
PLA-add_Ref	20.6	1.1	860 ± 16	274 ± 10
PLA-add_Lim	132.5	1.3	104 ± 11	105 ± 4
P200-5_Ref	20.6	1.1	840 ± 16	324 ± 15
P200-5_Lim	41.2	1.1	440 ± 25	222 ± 6
MCC-5_Ref	20.6	1.1	869 ± 29	286 ± 3
MCC-5_Lim	41.2	1.1	429 ± 8	224 ± 3

Ref = reference spinning condition used for direct comparison among formulations; Lim = highest stable draw condition achieved without filament breakage under the fixed comparative protocol used in this study.

## Data Availability

The original contributions presented in this study are included in the article. Further inquiries can be directed to the corresponding author.
